# Role of Sirtuins in Linking Metabolic Syndrome with Depression

**DOI:** 10.3389/fncel.2016.00086

**Published:** 2016-03-31

**Authors:** Juhyun Song, Jongpil Kim

**Affiliations:** Department of Biomedical Engineering, Dongguk UniversitySeoul, South Korea

**Keywords:** sirtuins (SIRTs), depression, inflammation, neurotransmitter, synaptic dysfunction, metabolic syndrome

## Abstract

Depression is now widely regarded as a common disabling disorder that affects negatively the social functioning all over the world. Depression is associated with diverse phenomenon in brain such as neuroinflammation, synaptic dysfunction, and cognitive deficit. Recent studies reported that depression occurs by various metabolic changes, leading to metabolic syndrome. Sirtuins (SIRTs) are NAD^+^-dependent class III histone deacetylases, known to regulate diverse biological mechanism such as longevity, genomic stability, and inflammation. The modulation of sirtuin activity has been highlighted as a promising approach to reduce neurodegenerative processes. In this review, we summarize the recent discoveries regarding the potential relationship between SIRTs and depression caused by metabolic disorders (Mets). Ultimately, we suggest the possibility that SIRTs will be novel targets to alleviate neuropathogenesis induced by depression.

## Introduction

The prevalence of depression continues to rise all over the world and yearly prevalence rate is close to 10% (Kessler et al., 2003, 2005, 2011; de Souza and Hidalgo, 2012). According to the world health organization (WHO) ranks regarding depression, depression will be the second leading cause of mortality worldwide in 2030 (Lopez and Mathers, 2006). Moreover, the patients with depression showed decreased expression of synapse related genes, the loss of synapse in hippocampus (Duric et al., 2013) and dendritic atrophy associated with depression-like behaviors (Morales-Medina et al., 2013). Finally, 94% of patients suffering from depression experience cognitive impairment (Conradi et al., 2011) including impairment of executive functions, attention, memory and learning (Jaeger et al., 2006; Murrough et al., 2011; Etkin et al., 2013; Trivedi and Greer, 2014). For these reasons, the increase of patients with depression is an important issue in the view of economical and sociological aspects. The causes of depression are mainly genetic factors (Lohoff, 2010), aberrant inflammatory response (Miller et al., 2009; Dowlati et al., 2010; Harry and Kraft, 2012) and the insufficient level of neurotransmitters including cortisol (Miller et al., 1999), serotonin (Maes et al., 2011), acetylcholine (Picciotto et al., 2015) and dopamine (Nutt, 2008). Furthermore, current studies highlight that depression patients reportedly exhibit the positive correlation with metabolic syndrome including diabetes and obesity (Pan et al., 2012; Silva et al., 2012). People with metabolic disorders (Mets) have higher prevalence of depression compared to those without metabolic syndromes (Pan et al., 2012; Sekita et al., 2013). Sirtuins (SIRTs), which are known as the important metabolism regulator, were categorized seven isoforms (SIRT1–7) characterizing by different substrate and subcellular localization (Michan and Sinclair, 2007; Nakagawa and Guarente, 2011). All SIRTs have different length of N-and C- terminal extensions and play variable role in mammal (Schwer et al., 2002; Tennen et al., 2010). SIRTs existed in the nucleus (SIRT1, 6, 7), cytosol (SIRT2), and mitochondria (SIRT3, 4, 5; Morris et al., 2011; Donmez and Outeiro, 2013). Sirtuin’s expression increases in cells exposed to conditions of oxidative stress and DNA damage (Cohen et al., 2004; Rodgers et al., 2005). Especially, SIRTs modulate diverse biological mechanisms including oxidative damage, protein aggregation, and inflammatory responses associated with central nervous system (CNS) diseases (Han, 2009) and play protective roles in neuropathological condition (Paraiso et al., 2013). Interestingly, a current study suggested that the expression of SIRT1, 2 and 6 mRNA in blood cells was altered in patients with mood disorders such as depression (Abe et al., 2011). Also, hippocampal SIRT2 enhances the depressant like behaviors by regulating neurogenesis (Liu et al., 2015). Here, we summarized recent evidences that SIRTs is involved in depressive disorder and SIRTs contributes to improve the depressive symptoms associated pathological phenomenon in metabolic stress condition. Thus, our review suggests that SIRTs may be good candidate genes to ameliorate the depressive symptoms.

## Sirtuins and Inflammation in Depression

**Figure 1:**
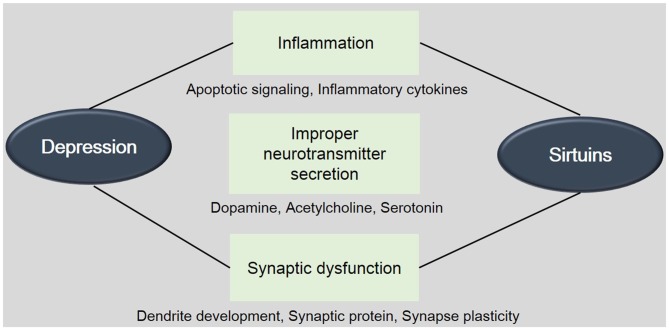
**The involvement between Sirtuins (SIRTs) and depression.** This schematic image shows the relevance between SIRTs and depression. The onset and progress of depression is related with the inflammation, improper neurotransmitter’s secretion, and synaptic dysfunction. This image indicates the involvement of SIRTs in these mechanisms. Mets: Metabolic disorders.

In a third of patients with depression, the serum and cerebrospinal fluid (CSF) concentrations of inflammatory markers in serum showed the elevation of pro-inflammatory factors such as interleukine (IL)-6, tumor necrosis factor (TNF)-α, and C-reactive protein compared to non-depressed patients (Raison et al., 2006; Dantzer et al., 2008; Dowlati et al., 2010; Liu et al., 2012). One study demonstrated that anti-depressants alleviate depressive state by suppressing the production of pro-inflammatory mediators such as IL-6 and nitric oxide (Hashioka et al., 2007). In addition, major depressive disorder were observed the reduction of natural killer cell cytotoxicity and T lymphocyte activity (Maes et al., 1991; Wong et al., 2008; Blume et al., 2011; Karg et al., 2011). In a clinical research, antidepressant treatment results in the enhancement of inflammatory response such as the reduction of IL-1 in depression patients and alleviates depressive symptoms (Hannestad et al., 2011). Based on various evidences, major depressive disorder leads to the inflammatory response including the activity of natural killer cell and lymphocyte, and the secretion of cytokines in brain. Hence, the decreased of inflammation may be a one of the solution for depression. SIRTs have known that they are involved in the inflammatory mechanisms through anti-apoptotic pathways. SIRT1 modulates the nuclear translocation of Forkhead box-containing protein O (FoxO) which is associated with the anti-apoptotic factor Bcl2 (Daitoku et al., 2004; Hsu et al., 2010). SIRT1 enhances cell survival in various stress conditions through the regulation of several substrates (Vaziri et al., 2001; Bordone et al., 2007). Several studies have mentioned the roles of SIRT1 as anti-apoptotic regulator that SIRT1 deacetylates the DNA repair factor (Jeong et al., 2007; Anekonda and Adamus, 2008; Mallick and D’Mello, 2014), and also inhibits p53 and NF-κB signaling (Cheng et al., 2003; Hernandez-Jimenez et al., 2013). Moreover, SIRT1 promotes growth of neurons and inhibits the death of neurons in CNS through mTOR signaling (Guo et al., 2011). SIRT1 also inhibits the release of pro-inflammatory cytokines in microglia (Ye et al., 2013). SIRT2 inhibits the inflammatory responses in CNS disorders by controlling the activation of microglia (Chen et al., 2015). SIRT2 prevents the excessive activation of microglia through NF-κB deacetylation (Pais et al., 2013) and regulate the cell cycle and the survival of microglia (Nie et al., 2014). SIRT3 protects cells against apoptotic cell death in oxidative stress by regulating anti-apoptotic signaling (Pellegrini et al., 2012; Chen et al., 2013) and by inhibiting the production of ROS (Kim et al., 2010). SIRT3 protects cortical neurons against H_2_O_2_ stimulated oxidative stress by regulating mitochondrial Ca^2+^ homeostasis and mitochondria dysfunction (Dai et al., 2014; Hu et al., 2014). Taken together, SIRTs have the pro-apoptotic effect via various pathways and is the regulator of cytokine secretion in CNS. Thus, we suggest that SIRTs may be strongly involved in the inflammatory response, leading to depression (Figure 1).

## Sirts and Neurotransmitter’S Insufficient Level in Depression

Depression has been reported that it is related with the alterations of neurotransmitters such as serotonin (5-HT), norepinephrine and dopamine (Nutt, 2008). In patients with depression, the level and activity of neurotransmitter were founded insufficiently compared to the normal subjects (Ruhe et al., 2007; Nutt, 2008). Considering the clinical research, monoaminergic modulators could improve the depressive symptoms in over 30–40% of all population (Roiser et al., 2012). In the state of depression, the level of serotonin, norepinephrine, and tyrosine known as the precursor of dopamine were insufficiency in the brain (Coppen, 1967) and in the blood of patients (Benkert et al., 1971; Antkiewicz-Michaluk et al., 2014). Also, the activity and level of dopamine associated with attention (Nutt, 2008) is dysregulated in major depressive disorders (Delgado, 2000; Dailly et al., 2004). In patients with major depressive disorders, the decreased level of GABA has been reported in plasma, CSF, and cortex neurons in comparison with the normal subjects (Rajkowska et al., 2007; Maciag et al., 2010). According to knockout mice model studies, the changes in 5-HT1B receptor expression and signaling were observed in the depression model (Lanfumey and Hamon, 2004; Lanfumey et al., 2008; Fakhoury, 2015). The lack of neurotransmitters subsequently alters the second messenger response in cells (Shimon et al., 1997; Coupland et al., 2005). Furthermore, several studies founded that the density of postsynaptic 5-HT receptors is markedly attenuated in patients with depression (Bhagwagar et al., 2004; Drevets et al., 2007). Recent studies demonstrated that SIRT1 affects the levels of neurotransmitter by modulating the mono-amine oxidases MAO-A promoter (Libert et al., 2011) and finally plays a beneficial role in the anxiety and depression (Nordquist and Oreland, 2010). The activation of SIRT1 also contributes to the regulation of GABA secretion (Prud’Homme et al., 2014). In addition, SIRT2 is involved in motor dysfunction in patients with neurodegenerative disease by decreasing dopamine content in the brain striatum (Wang et al., 2015). SIRT4 has been reported that it could modulate glutamate uptake in CNS (Shih et al., 2014).

In addition, depression is influenced by reduced levels and activity of acetylcholine (Caspi et al., 2003; Meerson et al., 2010). Current study demonstrated that mood and anxiety were regulated by acetylcholine pathway (Picciotto et al., 2015). Several studies suggested that SIRT1 could involve in acetylcholine receptor expression in brain (Huang et al., 2011) and modulates choline’s expression (Gareri et al., 2015). Taken together, SIRTs is involved in the level of neurotransmitters in CNS based on recent evidences. Hence, we suggest that SIRTs should be more highlighted the role of it regarding neurotransmitters in depression brain (Figure 1).

## Sirtuins and Synaptic Dysfunction in Depression

A loss of synaptic plasticity is commonly observed in patients with depression (Raison et al., 2006; Martinowich et al., 2007). Current clinic study demonstrated that the synaptic dysfunction is strongly influenced by depressive emotional states (McGaugh, 2000; Banasr and Duman, 2007; Kauer and Malenka, 2007; Pittenger and Duman, 2008; Russo and Nestler, 2013). Stress, leading to depression triggers the alteration of presynaptic glutamate secretion and postsynaptic glutamate receptor expression (Yuen et al., 2009). Depression also affects the dendrite spines and finally memory acquisition and long term plasticity (Alvarez and Sabatini, 2007). Recent study reported that SIRT1 affects dendritic development in hippocampal neurons (Braidy et al., 2012). In SIRT1 knockout mice, dendrite branching and branch length of neuron decreased compared to the normal brain (Michan et al., 2010) and impaired synapse plasticity was founded in the hippocampus (Gao et al., 2010). The activity of SIRT1 in dendritic development is related with the Rho GTPases and the ROCK signaling in hippocampal neuron (Negishi and Katoh, 2002; Codocedo et al., 2012). Several studies indicate that the activity of SIRT1 protects the several synaptic proteins in various neurodegenerative diseases (Duan and Mattson, 1999; Patel et al., 2005; Kim et al., 2007). Collectively, SIRTs are associated with the synaptic dysfunction and may contribute to the improvement of synapse plasticity in depression (Figure 1).

## Sirtuins and Metabolic Syndrome with Depression

**Figure 2:**
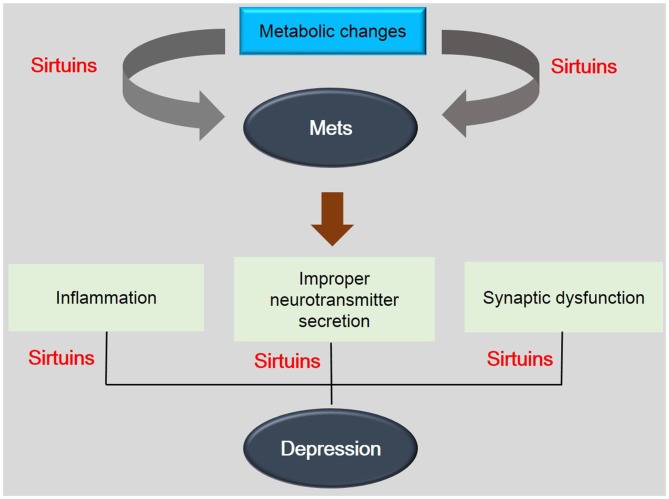
**The association between depression and metabolic diseases.** This schematic image shows the association between depression and metabolic diseases. SIRTs are involved in the pathogenesis of metabolic disease by metabolic changes and also are related with depression related phenomenon. This image indicates the importance of SIRTs in mechanisms between depression and metabolic diseases. Mets: Metabolic disorders.

Recently, the elevated level of metabolic factors including blood pressure, cholesterol, C-reactive protein leading to T2DM or obesity (Ali et al., 2006; Barnard et al., 2006) has been considered as the higher risk for depressive symptoms (Hamer et al., 2012; Rotella and Mannucci, 2013). High glucose and insulin resistance in T2DM affect negatively the brain (van Duinkerken et al., 2012a; Antenor-Dorsey et al., 2013; Reijmer et al., 2013) because it aggravates the functional dysconnectivity of brain (Geissler et al., 2003; Sahin et al., 2008; Musen et al., 2012; van Duinkerken et al., 2012b). Hyperglycemia induces the dysregulation of hypothalamic pituitary-adrenal axis and dysregulation of monoaminergic system (Zanoveli et al., 2015). Several studies reported that the patients with obesity and depression have more frequency in comparison with the general population (McIntyre et al., 2007; Blaine, 2008; Luppino et al., 2010; Levitan et al., 2012; Toups et al., 2013). According on previous published evidences, SIRT1 is strongly related with neuropathgenesis caused by T2DM and obesity. SIRT1 has been reported that it plays a cardinal role in glucose metabolism and insulin signaling activation (Guarente, 2006; Barzilai et al., 2012; Wang et al., 2012; Mortuza et al., 2013; Silvestre et al., 2014). The regulation of glucose metabolism is an important issue for anti-aging according several evidences (Colman et al., 2009; Smith et al., 2010). The regulation of glucose metabolism has been demonstrated to suppress against onset age related diseases (Colman et al., 2009; Smith et al., 2010) such as the T2DM and cardiovascular disease (Hammer et al., 2008; Marchal et al., 2012) and especially is associated with the activity of AMPK and SIRT1 related with the NAD^+^ biosynthetic activity (Yang et al., 2007) and SIRT6 associated with the regulation of insulin mediated signaling (Xiao et al., 2010). SIRTs improved insulin secretion and glucose homeostasis (Revollo et al., 2007; Caron et al., 2014) via increasing NAD^+^ levels (Schenk et al., 2011). Based on the study using SIRT6 (−/−) animals, the deficiency of SIRT6 showed the changes of blood glucose level (Xiao et al., 2010). In addition, in neurons, SIRT1 signaling modulates peroxisome proliferator-activated receptor γ coactivator-1 (PGC-1α) activity and subsequently mitochondrial dysfunction (Chowdhury et al., 2011). SIRT2 also has been reported that it is target for diabetes (Nerurkar and Nerurkar, 2008). Taken together, SIRTs family is associated with the metabolic diseases including the T2DM and obesity and affect neuropathogenesis caused by these disorders (Hamer et al., 2012; Rotella and Mannucci, 2013). Taken together, SIRTs is involved in the progress of depressive symptoms caused by metabolic disease (Figure 2). Considering sirtuin’s neuroprotective effects including anti-inflammatory effect, regulation of neurotransmitter production, and reduction of synaptic dysfunction, SIRTs could be a crucial target to ameliorate depressive pathology by metabolic alterations.

## Conclusions and Future Perspectives

In conclusion, we suggest that SIRTs are promising therapeutic targets to alleviate depression pathology in four possibilities: (1) SIRTs may control the inflammatory response in depression; (2) SIRTs might regulate the insufficient level of neurotransmitters in depression; (3) SIRTs may improve the synaptic dysfunction caused by depression; and (4) sirtuis may alleviate the cognitive decline caused by depression. As society ages, people suffer from metabolic syndrome such as diabetes, obesity, and cardiovascular disease and subsequently they suffer from depression by metabolic changes. We should investigate to search the therapeutic solution. SIRTs is the metabolism related genes and also ameliorates depression related pathogenesis (Abe et al., 2011; Ferland et al., 2013; Krogh et al., 2014). SIRTs may be a good target to modulate various pathogenesis in depression related with metabolic diseases. Although sirtuin’s mechanisms in depression is fully not understood until now, researchers continuously have investigated the novel role of SIRTs such as the regulation of microRNAs by SIRTs (Rao et al., 2013; Deng et al., 2014; Rodriguez-Ortiz et al., 2014). Thus, we propose the necessity of further studies regarding sirtuin’s role, suggesting that manipulation of siutuins may be a therapeutic solution for depression.

## Author Contributions

JS wrote the preliminary draft and revised details of the manuscript. JK revised all manuscript in detail.

## Conflict of Interest Statement

The authors declare that the research was conducted in the absence of any commercial or financial relationships that could be construed as a potential conflict of interest.

## References

[B1] Abe N. Uchida S. Otsuki K. Hobara T. Yamagata H. Higuchi F.. (2011). Altered sirtuin deacetylase gene expression in patients with a mood disorder. J. Psychiatr. Res. 45, 1106–1112. 10.1016/j.jpsychires.2011.01.016 21349544

[B2] Ali S. Stone M. A. Peters J. L. Davies M. J. Khunti K. (2006). The prevalence of co-morbid depression in adults with Type 2 diabetes: a systematic review and meta-analysis. Diabet. Med. 23, 1165–1173. 10.1111/j.1464-5491.2006.01943.x 17054590

[B3] Alvarez V. A. Sabatini B. L. (2007). Anatomical and physiological plasticity of dendritic spines. Annu. Rev. Neurosci. 30, 79–97. 10.1146/annurev.neuro.30.051606.094222 17280523

[B4] Anekonda T. S. Adamus G. (2008). Resveratrol prevents antibody-induced apoptotic death of retinal cells through upregulation of Sirt1 and Ku70. BMC Res. Notes 1:122. 10.1186/1756-0500-1-122 19046449 PMC2633309

[B5] Antenor-Dorsey J. A. Meyer E. Rutlin J. Perantie D. C. White N. H. Arbelaez A. M.. (2013). White matter microstructural integrity in youth with type 1 diabetes. Diabetes 62, 581–589. 10.2337/db12-0696 23139349 PMC3554385

[B6] Antkiewicz-Michaluk L. Wasik A. Mozdzen E. Romanska I. Michaluk J. (2014). Antidepressant-like effect of tetrahydroisoquinoline amines in the animal model of depressive disorder induced by repeated administration of a low dose of reserpine: behavioral and neurochemical studies in the rat. Neurotox. Res. 26, 85–98. 10.1007/s12640-013-9454-8 24407488 PMC4035545

[B7] Banasr M. Duman R. S. (2007). Regulation of neurogenesis and gliogenesis by stress and antidepressant treatment. CNS Neurol. Disord. Drug Targets 6, 311–320. 10.2174/187152707783220929 18045159

[B8] Barnard K. D. Skinner T. C. Peveler R. (2006). The prevalence of co-morbid depression in adults with Type 1 diabetes: systematic literature review. Diabet. Med. 23, 445–448. 10.1111/j.1464-5491.2006.01814.x 16620276

[B9] Barzilai N. Huffman D. M. Muzumdar R. H. Bartke A. (2012). The critical role of metabolic pathways in aging. Diabetes 61, 1315–1322. 10.2337/db11-1300 22618766 PMC3357299

[B10] Benkert O. Renz A. Marano C. Matussek N. (1971). Altered tyrosine daytime plasma levels in endogenous depressive patients. Arch. Gen. Psychiatry 25, 359–363. 10.1001/archpsyc.1971.01750160071013 5116991

[B11] Bhagwagar Z. Rabiner E. A. Sargent P. A. Grasby P. M. Cowen P. J. (2004). Persistent reduction in brain serotonin1A receptor binding in recovered depressed men measured by positron emission tomography with [11C]WAY-100635. Mol. Psychiatry 9, 386–392. 10.1038/sj.mp.4001401 15042104

[B12] Blaine B. (2008). Does depression cause obesity? A meta-analysis of longitudinal studies of depression and weight control. J. Health Psychol. 13, 1190–1197. 10.1177/1359105308095977 18987092

[B13] Blume J. Douglas S. D. Evans D. L. (2011). Immune suppression and immune activation in depression. Brain Behav. Immun. 25, 221–229. 10.1016/j.bbi.2010.10.008 20955778 PMC3025086

[B14] Bordone L. Cohen D. Robinson A. Motta M. C. van Veen E. Czopik A.. (2007). SIRT1 transgenic mice show phenotypes resembling calorie restriction. Aging Cell 6, 759–767. 10.1111/j.1474-9726.2007.00335.x 17877786

[B15] Braidy N. Jayasena T. Poljak A. Sachdev P. S. (2012). Sirtuins in cognitive ageing and Alzheimer’s disease. Curr. Opin. Psychiatry 25, 226–230. 10.1097/yco.0b013e32835112c1 22327552

[B16] Caron A. Z. He X. Mottawea W. Seifert E. L. Jardine K. Dewar-Darch D.. (2014). The SIRT1 deacetylase protects mice against the symptoms of metabolic syndrome. FASEB J. 28, 1306–1316. 10.1096/fj.13-243568 24297700

[B17] Caspi A. Sugden K. Moffitt T. E. Taylor A. Craig I. W. Harrington H.. (2003). Influence of life stress on depression: moderation by a polymorphism in the 5-HTT gene. Science 301, 386–389. 10.1126/science.1083968 12869766

[B18] Chen C. J. Fu Y. C. Yu W. Wang W. (2013). SIRT3 protects cardiomyocytes from oxidative stress-mediated cell death by activating NF-κB. Biochem. Biophys. Res. Commun. 430, 798–803. 10.1016/j.bbrc.2012.11.066 23201401

[B19] Chen H. Wu D. Ding X. Ying W. (2015). SIRT2 is required for lipopolysaccharide-induced activation of BV2 microglia. Neuroreport 26, 88–93. 10.1097/wnr.0000000000000305 25536118

[B20] Cheng H. L. Mostoslavsky R. Saito S. Manis J. P. Gu Y. Patel P.. (2003). Developmental defects and p53 hyperacetylation in Sir2 homolog (SIRT1)-deficient mice. Proc. Natl. Acad. Sci. U S A 100, 10794–10799. 10.1073/pnas.1934713100 12960381 PMC196882

[B21] Chowdhury S. K. Dobrowsky R. T. Fernyhough P. (2011). Nutrient excess and altered mitochondrial proteome and function contribute to neurodegeneration in diabetes. Mitochondrion 11, 845–854. 10.1016/j.mito.2011.06.007 21742060 PMC3375692

[B22] Codocedo J. F. Allard C. Godoy J. A. Varela-Nallar L. Inestrosa N. C. (2012). SIRT1 regulates dendritic development in hippocampal neurons. PLoS One 7:e47073. 10.1371/journal.pone.0047073 23056585 PMC3464248

[B23] Cohen H. Y. Miller C. Bitterman K. J. Wall N. R. Hekking B. Kessler B.. (2004). Calorie restriction promotes mammalian cell survival by inducing the SIRT1 deacetylase. Science 305, 390–392. 10.1126/science.1099196 15205477

[B24] Colman R. J. Anderson R. M. Johnson S. C. Kastman E. K. Kosmatka K. J. Beasley T. M.. (2009). Caloric restriction delays disease onset and mortality in rhesus monkeys. Science 325, 201–204. 10.1126/science.1173635 19590001 PMC2812811

[B25] Conradi H. J. Ormel J. de Jonge P. (2011). Presence of individual (residual) symptoms during depressive episodes and periods of remission: a 3-year prospective study. Psychol. Med. 41, 1165–1174. 10.1017/s0033291710001911 20932356

[B26] Coppen A. (1967). The biochemistry of affective disorders. Br. J. Psychiatry 113, 1237–1264. 4169954 10.1192/bjp.113.504.1237

[B27] Coupland N. J. Ogilvie C. J. Hegadoren K. M. Seres P. Hanstock C. C. Allen P. S. (2005). Decreased prefrontal Myo-inositol in major depressive disorder. Biol. Psychiatry 57, 1526–1534. 10.1016/j.biopsych.2005.02.027 15953489

[B28] Dai S. H. Chen T. Wang Y. H. Zhu J. Luo P. Rao W.. (2014). Sirt3 protects cortical neurons against oxidative stress via regulating mitochondrial Ca2+ and mitochondrial biogenesis. Int. J. Mol. Sci. 15, 14591–14609. 10.3390/ijms150814591 25196599 PMC4159870

[B29] Dailly E. Chenu F. Renard C. E. Bourin M. (2004). Dopamine, depression and antidepressants. Fundam. Clin. Pharmacol. 18, 601–607. 10.1111/j.1472-8206.2004.00287.x 15548230

[B30] Daitoku H. Hatta M. Matsuzaki H. Aratani S. Ohshima T. Miyagishi M.. (2004). Silent information regulator 2 potentiates Foxo1-mediated transcription through its deacetylase activity. Proc. Natl. Acad. Sci. U S A. 101, 10042–10047. 10.1073/pnas.0400593101 15220471 PMC454161

[B31] Dantzer R. O’Connor J. C. Freund G. G. Johnson R. W. Kelley K. W. (2008). From inflammation to sickness and depression: when the immune system subjugates the brain. Nat. Rev. Neurosci. 9, 46–56. 10.1038/nrn2297 18073775 PMC2919277

[B32] de Souza C. M. Hidalgo M. P. (2012). World Health Organization 5-item well-being index: validation of the Brazilian Portuguese version. Eur. Arch. Psychiatry Clin. Neurosci. 262, 239–244. 10.1007/s00406-011-0255-x 21912931

[B33] Delgado P. L. (2000). Depression: the case for a monoamine deficiency. J. Clin. Psychiatry 61 Suppl 6, 7–11. 10775018

[B34] Deng S. Zhu S. Wang B. Li X. Liu Y. Qin Q.. (2014). Chronic pancreatitis and pancreatic cancer demonstrate active epithelial-mesenchymal transition profile, regulated by miR-217-SIRT1 pathway. Cancer Lett. 355, 184–191. 10.1016/j.canlet.2014.08.007 25172416

[B35] Donmez G. Outeiro T. F. (2013). SIRT1 and SIRT2: emerging targets in neurodegeneration. EMBO Mol. Med. 5, 344–352. 10.1002/emmm.201302451 23417962 PMC3598076

[B36] Dowlati Y. Herrmann N. Swardfager W. Liu H. Sham L. Reim E. K.. (2010). A meta-analysis of cytokines in major depression. Biol. Psychiatry 67, 446–457. 10.1016/j.biopsych.2009.09.033 20015486

[B37] Drevets W. C. Thase M. E. Moses-Kolko E. L. Price J. Frank E. Kupfer D. J.. (2007). Serotonin-1A receptor imaging in recurrent depression: replication and literature review. Nucl. Med. Biol. 34, 865–877. 10.1016/j.nucmedbio.2007.06.008 17921037 PMC2702715

[B38] Duan W. Mattson M. P. (1999). Dietary restriction and 2-deoxyglucose administration improve behavioral outcome and reduce degeneration of dopaminergic neurons in models of Parkinson’s disease. J. Neurosci. Res. 57, 195–206. 10.1002/(sici)1097-4547(19990715)57:2<195::aid-jnr5>3.0.co;2-p 10398297

[B39] Duric V. Banasr M. Stockmeier C. A. Simen A. A. Newton S. S. Overholser J. C.. (2013). Altered expression of synapse and glutamate related genes in post-mortem hippocampus of depressed subjects. Int. J. Neuropsychopharmacol. 16, 69–82. 10.1017/s1461145712000016 22339950 PMC3414647

[B40] Etkin A. Gyurak A. O’Hara R. (2013). A neurobiological approach to the cognitive deficits of psychiatric disorders. Dialogues Clin. Neurosci. 15, 419–429. 24459409 10.31887/DCNS.2013.15.4/aetkin PMC3898680

[B41] Fakhoury M. (2015). Revisiting the serotonin hypothesis: implications for major depressive disorders. Mol. Neurobiol. 10.1007/s12035-015-9152-z [Epub ahead of print]. 25823514

[B42] Ferland C. L. Hawley W. R. Puckett R. E. Wineberg K. Lubin F. D. Dohanich G. P.. (2013). Sirtuin activity in dentate gyrus contributes to chronic stress-induced behavior and extracellular signal-regulated protein kinases 1 and 2 cascade changes in the hippocampus. Biol. Psychiatry 74, 927–935. 10.1016/j.biopsych.2013.07.029 24011821 PMC4142505

[B43] Gao L. Chao L. Chao J. (2010). A novel signaling pathway of tissue kallikrein in promoting keratinocyte migration: activation of proteinase-activated receptor 1 and epidermal growth factor receptor. Exp. Cell Res. 316, 376–389. 10.1016/j.yexcr.2009.10.022 19879874 PMC2812679

[B44] Gareri P. Castagna A. Cotroneo A. M. Putignano S. De Sarro G. Bruni A. C. (2015). The role of citicoline in cognitive impairment: pharmacological characteristics, possible advantages and doubts for an old drug with new perspectives. Clin. Interv. Aging 10, 1421–1429. 10.2147/cia.s87886 26366063 PMC4562749

[B45] Geissler A. Frund R. Scholmerich J. Feuerbach S. Zietz B. (2003). Alterations of cerebral metabolism in patients with diabetes mellitus studied by proton magnetic resonance spectroscopy. Exp. Clin. Endocrinol. Diabetes. 111, 421–427. 10.1055/s-2003-44289 14614649

[B46] Guarente L. (2006). Sirtuins as potential targets for metabolic syndrome. Nature 444, 868–874. 10.1038/nature05486 17167475

[B47] Guo W. Qian L. Zhang J. Zhang W. Morrison A. Hayes P.. (2011). Sirt1 overexpression in neurons promotes neurite outgrowth and cell survival through inhibition of the mTOR signaling. J. Neurosci. Res. 89, 1723–1736. 10.1002/jnr.22725 21826702

[B48] Hamer M. Batty G. D. Kivimaki M. (2012). Risk of future depression in people who are obese but metabolically healthy: the English longitudinal study of ageing. Mol. Psychiatry 17, 940–945. 10.1038/mp.2012.30 22525487 PMC3428506

[B49] Hammer S. Snel M. Lamb H. J. Jazet I. M. van der Meer R. W. Pijl H.. (2008). Prolonged caloric restriction in obese patients with type 2 diabetes mellitus decreases myocardial triglyceride content and improves myocardial function. J. Am. Coll. Cardiol. 52, 1006–1012. 10.1016/j.jacc.2008.04.068 18786482

[B50] Han S. H. (2009). Potential role of sirtuin as a therapeutic target for neurodegenerative diseases. J. Clin. Neurol. 5, 120–125. 10.3988/jcn.2009.5.3.120 19826562 PMC2760716

[B51] Hannestad J. DellaGioia N. Bloch M. (2011). The effect of antidepressant medication treatment on serum levels of inflammatory cytokines: a meta-analysis. Neuropsychopharmacology 36, 2452–2459. 10.1038/npp.2011.132 21796103 PMC3194072

[B52] Harry G. J. Kraft A. D. (2012). Microglia in the developing brain: a potential target with lifetime effects. Neurotoxicology 33, 191–206. 10.1016/j.neuro.2012.01.012 22322212 PMC3299893

[B53] Hashioka S. Klegeris A. Monji A. Kato T. Sawada M. McGeer P. L.. (2007). Antidepressants inhibit interferon-gamma-induced microglial production of IL-6 and nitric oxide. Exp. Neurol. 206, 33–42. 10.1016/j.expneurol.2007.03.022 17481608

[B54] Hernandez-Jimenez M. Hurtado O. Cuartero M. I. Ballesteros I. Moraga A. Pradillo J. M.. (2013). Silent information regulator 1 protects the brain against cerebral ischemic damage. Stroke 44, 2333–2337. 10.1161/strokeaha.113.001715 23723308

[B55] Hsu C. P. Zhai P. Yamamoto T. Maejima Y. Matsushima S. Hariharan N.. (2010). Silent information regulator 1 protects the heart from ischemia/reperfusion. Circulation 122, 2170–2182. 10.1161/circulationaha.110.958033 21060073 PMC3003297

[B56] Hu W. Guan L. S. Dang X. B. Ren P. Y. Zhang Y. L. (2014). Small-molecule inhibitors at the PSD-95/nNOS interface attenuate MPP^+^-induced neuronal injury through Sirt3 mediated inhibition of mitochondrial dysfunction. Neurochem. Int. 79, 57–64. 10.1016/j.neuint.2014.10.005 25452082

[B57] Huang P. S. Son J. H. Abbott L. C. Winzer-Serhan U. H. (2011). Regulated expression of neuronal SIRT1 and related genes by aging and neuronal β2-containing nicotinic cholinergic receptors. Neuroscience 196, 189–202. 10.1016/j.neuroscience.2011.09.007 21939740

[B58] Jaeger J. Berns S. Uzelac S. Davis-Conway S. (2006). Neurocognitive deficits and disability in major depressive disorder. Psychiatry Res. 145, 39–48. 10.1016/j.psychres.2005.11.011 17045658

[B59] Jeong J. Juhn K. Lee H. Kim S. H. Min B. H. Lee K. M.. (2007). SIRT1 promotes DNA repair activity and deacetylation of Ku70. Exp. Mol. Med. 39, 8–13. 10.1038/emm.2007.2 17334224

[B60] Karg K. Burmeister M. Shedden K. Sen S. (2011). The serotonin transporter promoter variant (5-HTTLPR), stress and depression meta-analysis revisited: evidence of genetic moderation. Arch. Gen. Psychiatry 68, 444–454. 10.1001/archgenpsychiatry.2010.189 21199959 PMC3740203

[B61] Kauer J. A. Malenka R. C. (2007). Synaptic plasticity and addiction. Nat. Rev. Neurosci. 8, 844–858. 10.1038/nrn2234 17948030

[B62] Kessler R. C. Berglund P. Demler O. Jin R. Koretz D. Merikangas K. R.. (2003). The epidemiology of major depressive disorder: results from the national comorbidity survey replication (NCS-R). JAMA 289, 3095–3105. 10.1001/jama.289.23.3095 12813115

[B63] Kessler R. C. Chiu W. T. Demler O. Merikangas K. R. Walters E. E. (2005). Prevalence, severity and comorbidity of 12-month DSM-IV disorders in the national comorbidity survey replication. Arch. Gen. Psychiatry 62, 617–627. 10.1001/archpsyc.62.6.617 15939839 PMC2847357

[B64] Kessler R. C. Ormel J. Petukhova M. McLaughlin K. A. Green J. G. Russo L. J.. (2011). Development of lifetime comorbidity in the World Health Organization world mental health surveys. Arch. Gen. Psychiatry 68, 90–100. 10.1001/archgenpsychiatry.2010.180 21199968 PMC3057480

[B65] Kim D. Nguyen M. D. Dobbin M. M. Fischer A. Sananbenesi F. Rodgers J. T.. (2007). SIRT1 deacetylase protects against neurodegeneration in models for Alzheimer’s disease and amyotrophic lateral sclerosis. EMBO J. 26, 3169–3179. 10.1038/sj.emboj.7601758 17581637 PMC1914106

[B66] Kim H. S. Patel K. Muldoon-Jacobs K. Bisht K. S. Aykin-Burns N. Pennington J. D.. (2010). SIRT3 is a mitochondria-localized tumor suppressor required for maintenance of mitochondrial integrity and metabolism during stress. Cancer Cell 17, 41–52. 10.1016/j.ccr.2009.11.023 20129246 PMC3711519

[B67] Krogh J. Benros M. E. Jorgensen M. B. Vesterager L. Elfving B. Nordentoft M. (2014). The association between depressive symptoms, cognitive function and inflammation in major depression. Brain Behav. Immun. 35, 70–76. 10.1016/j.bbi.2013.08.014 24016864

[B68] Lanfumey L. Hamon M. (2004). 5-HT1 receptors. Curr. Drug Targets CNS Neurol. Disord. 3, 1–10. 10.2174/1568007043482570 14965240

[B69] Lanfumey L. Mongeau R. Cohen-Salmon C. Hamon M. (2008). Corticosteroid-serotonin interactions in the neurobiological mechanisms of stress-related disorders. Neurosci. Biobehav. Rev. 32, 1174–1184. 10.1016/j.neubiorev.2008.04.006 18534678

[B70] Levitan R. D. Davis C. Kaplan A. S. Arenovich T. Phillips D. I. Ravindran A. V. (2012). Obesity comorbidity in unipolar major depressive disorder: refining the core phenotype. J. Clin. Psychiatry 73, 1119–1124. 10.4088/jcp.11m07394 22687640

[B71] Libert S. Pointer K. Bell E. L. Das A. Cohen D. E. Asara J. M.. (2011). SIRT1 activates MAO-A in the brain to mediate anxiety and exploratory drive. Cell 147, 1459–1472. 10.1016/j.cell.2011.10.054 22169038 PMC3443638

[B72] Liu R. Dang W. Du Y. Zhou Q. Jiao K. Liu Z. (2015). SIRT2 is involved in the modulation of depressive behaviors. Sci. Rep. 5: 8415. 10.1038/srep08415 25672834 PMC4325337

[B73] Liu Y. Ho R. C. Mak A. (2012). Interleukin (IL)-6, tumour necrosis factor alpha (TNF-α) and soluble interleukin-2 receptors (sIL-2R) are elevated in patients with major depressive disorder: a meta-analysis and meta-regression. J. Affect Disord. 139, 230–239. 10.1016/j.jad.2011.08.003 21872339

[B74] Lohoff F. W. (2010). Overview of the genetics of major depressive disorder. Curr. Psychiatry Rep. 12, 539–546. 10.1007/s11920-010-0150-6 20848240 PMC3077049

[B75] Lopez A. D. Mathers C. D. (2006). Measuring the global burden of disease and epidemiological transitions. 2002–2030. Ann. Trop. Med. Parasitol. 100, 481–499. 10.1179/136485906x97417 16899150

[B76] Luppino F. S. de Wit L. M. Bouvy P. F. Stijnen T. Cuijpers P. Penninx B. W.. (2010). Overweight, obesity and depression: a systematic review and meta-analysis of longitudinal studies. Arch. Gen. Psychiatry 67, 220–229. 10.1001/archgenpsychiatry.2010.2 20194822

[B77] Maciag D. Hughes J. O’Dwyer G. Pride Y. Stockmeier C. A. Sanacora G.. (2010). Reduced density of calbindin immunoreactive GABAergic neurons in the occipital cortex in major depression: relevance to neuroimaging studies. Biol. Psychiatry 67, 465–470. 10.1016/j.biopsych.2009.10.027 20004363 PMC2823848

[B78] Maes M. Bosmans E. Suy E. Vandervorst C. DeJonckheere C. Raus J. (1991). Depression-related disturbances in mitogen-induced lymphocyte responses and interleukin 1β and soluble interleukin-2 receptor production. Acta Psychiatr. Scand. 84, 379–386. 10.1111/j.1600-0447.1991.tb03163.x 1746291

[B79] Maes M. Leonard B. E. Myint A. M. Kubera M. Verkerk R. (2011). The new ′5-HT’ hypothesis of depression: cell-mediated immune activation induces indoleamine 2,3-dioxygenase, which leads to lower plasma tryptophan and an increased synthesis of detrimental tryptophan catabolites (TRYCATs), both of which contribute to the onset of depression. Prog. Neuropsychopharmacol. Biol. Psychiatry. 35, 702–721. 10.1016/j.pnpbp.2010.12.017 21185346

[B80] Mallick S. D’Mello S. R. (2014). JAZ (Znf346), a SIRT1-interacting protein, protects neurons by stimulating p21 (WAF/CIP1) protein expression. J. Biol. Chem. 289, 35409–35420. 10.1074/jbc.m114.597575 25331946 PMC4271226

[B81] Marchal J. Blanc S. Epelbaum J. Aujard F. Pifferi F. (2012). Effects of chronic calorie restriction or dietary resveratrol supplementation on insulin sensitivity markers in a primate, Microcebus murinus. PloS One 7:e34289. 10.1371/journal.pone.0034289 22479589 PMC3316613

[B82] Martinowich K. Manji H. Lu B. (2007). New insights into BDNF function in depression and anxiety. Nat. Neurosci. 10, 1089–1093. 10.1038/nn1971 17726474

[B83] McGaugh J. L. (2000). Memory—a century of consolidation. Science 287, 248–251. 10.1126/science.287.5451.248 10634773

[B84] McIntyre R. S. Soczynska J. K. Konarski J. Z. Woldeyohannes H. O. Law C. W. Miranda A.. (2007). Should depressive syndromes be reclassified as “metabolic syndrome type II”? Ann. Clin. Psychiatry 19, 257–264. 10.1080/10401230701653377 18058283

[B85] Meerson A. Cacheaux L. Goosens K. A. Sapolsky R. M. Soreq H. Kaufer D. (2010). Changes in brain MicroRNAs contribute to cholinergic stress reactions. J. Mol. Neurosci. 40, 47–55. 10.1007/s12031-009-9252-1 19711202 PMC2807969

[B86] Michan S. Sinclair D. (2007). Sirtuins in mammals: insights into their biological function. Biochem. J. 404, 1–13. 10.1042/bj20070140 17447894 PMC2753453

[B87] Michan S. Li Y. Chou M. M. Parrella E. Ge H. Long J. M.. (2010). SIRT1 is essential for normal cognitive function and synaptic plasticity. J. Neurosci. 30, 9695–9707. 10.1523/jneurosci.0027-10.2010 20660252 PMC2921958

[B88] Miller A. H. Maletic V. Raison C. L. (2009). Inflammation and its discontents: the role of cytokines in the pathophysiology of major depression. Biol. Psychiatry 65, 732–741. 10.1016/j.biopsych.2008.11.029 19150053 PMC2680424

[B89] Miller A. H. Pariante C. M. Pearce B. D. (1999). Effects of cytokines on glucocorticoid receptor expression and function. Glucocorticoid resistance and relevance to depression. Adv. Exp. Med. Biol. 461, 107–116. 10.1007/978-0-585-37970-8_7 10442170

[B90] Morales-Medina J. C. Juarez I. Venancio-Garcia E. Cabrera S. N. Menard C. Yu W.. (2013). Impaired structural hippocampal plasticity is associated with emotional and memory deficits in the olfactory bulbectomized rat. Neuroscience 236, 233–243. 10.1016/j.neuroscience.2013.01.037 23357118

[B91] Morris K. C. Lin H. W. Thompson J. W. Perez-Pinzon M. A. (2011). Pathways for ischemic cytoprotection: role of sirtuins in caloric restriction, resveratrol and ischemic preconditioning. J. Cereb. Blood. Flow. Metab. 31, 1003–1019. 10.1038/jcbfm.2010.229 21224864 PMC3070983

[B92] Mortuza R. Chen S. Feng B. Sen S. Chakrabarti S. (2013). High glucose induced alteration of SIRTs in endothelial cells causes rapid aging in a p300 and FOXO regulated pathway. PLoS One 8:e54514. 10.1371/journal.pone.0054514 23342163 PMC3546959

[B93] Murrough J. W. Iacoviello B. Neumeister A. Charney D. S. Iosifescu D. V. (2011). Cognitive dysfunction in depression: neurocircuitry and new therapeutic strategies. Neurobiol. Learn. Mem. 96, 553–563. 10.1016/j.nlm.2011.06.006 21704176

[B94] Musen G. Jacobson A. M. Bolo N. R. Simonson D. C. Shenton M. E. McCartney R. L.. (2012). Resting-state brain functional connectivity is altered in type 2 diabetes. Diabetes 61, 2375–2379. 10.2337/db11-1669 22664957 PMC3425418

[B95] Nakagawa T. Guarente L. (2011). Sirtuins at a glance. J. Cell Sci. 124, 833–838. 10.1242/jcs.081067 21378304 PMC3048886

[B96] Negishi M. Katoh H. (2002). Rho family GTPases as key regulators for neuronal network formation. J. Biochem. 132, 157–166. 10.1093/oxfordjournals.jbchem.a003205 12153710

[B97] Nerurkar P. V. Nerurkar V. R. (2008). Respected Sir(2): magic target for diabetes. Cellscience 4, 82–96. 20577646 PMC2890243

[B98] Nie H. Li Y. Wang C. Chen X. Liu B. Wu D.. (2014). SIRT2 plays a key role in both cell cycle regulation and cell survival of BV2 microglia. Int. J. Physiol. Pathophysiol. Pharmacol. 6, 166–171. 25349639 PMC4208737

[B99] Nordquist N. Oreland L. (2010). Serotonin, genetic variability, behaviour and psychiatric disorders—a review. Ups. J. Med. Sci. 115, 2–10. 10.3109/03009730903573246 20187845 PMC2853349

[B100] Nutt D. J. (2008). Relationship of neurotransmitters to the symptoms of major depressive disorder. J. Clin. Psychiatry 69 Suppl E1, 4–7. 10.1007/springerreference_116852 18494537

[B101] Pais T. F. Szego E. M. Marques O. Miller-Fleming L. Antas P. Guerreiro P.. (2013). The NAD-dependent deacetylase sirtuin 2 is a suppressor of microglial activation and brain inflammation. EMBO J. 32, 2603–2616. 10.1038/emboj.2013.200 24013120 PMC3791374

[B102] Pan A. Keum N. Okereke O. I. Sun Q. Kivimaki M. Rubin R. R.. (2012). Bidirectional association between depression and metabolic syndrome: a systematic review and meta-analysis of epidemiological studies. Diabetes Care 35, 1171–1180. 10.2337/dc11-2055 22517938 PMC3329841

[B103] Paraiso A. F. Mendes K. L. Santos S. H. (2013). Brain activation of SIRT1: role in neuropathology. Mol. Neurobiol. 48, 681–689. 10.1007/s12035-013-8459-x 23615921

[B104] Patel N. V. Gordon M. N. Connor K. E. Good R. A. Engelman R. W. Mason J.. (2005). Caloric restriction attenuates Aβ-deposition in Alzheimer transgenic models. Neurobiol. Aging 26, 995–1000. 10.1016/j.neurobiolaging.2004.09.014 15748777

[B105] Pellegrini L. Pucci B. Villanova L. Marino M. L. Marfe G. Sansone L.. (2012). SIRT3 protects from hypoxia and staurosporine-mediated cell death by maintaining mitochondrial membrane potential and intracellular pH. Cell Death Differ. 19, 1815–1825. 10.1038/cdd.2012.62 22595756 PMC3469066

[B106] Picciotto M. R. Lewis A. S. van Schalkwyk G. I. Mineur Y. S. (2015). Mood and anxiety regulation by nicotinic acetylcholine receptors: A potential pathway to modulate aggression and related behavioral states. Neuropharmacology 96, 235–243. 10.1016/j.neuropharm.2014.12.028 25582289 PMC4486625

[B107] Pittenger C. Duman R. S. (2008). Stress, depression and neuroplasticity: a convergence of mechanisms. Neuropsychopharmacology 33, 88–109. 10.1038/sj.npp.1301574 17851537

[B108] Prud’Homme G. J. Glinka Y. Udovyk O. Hasilo C. Paraskevas S. Wang Q. (2014). GABA protects pancreatic beta cells against apoptosis by increasing SIRT1 expression and activity. Biochem. Biophys. Res. Commun. 452, 649–654. 10.1016/j.bbrc.2014.08.135 25193706

[B109] Raison C. L. Capuron L. Miller A. H. (2006). Cytokines sing the blues: inflammation and the pathogenesis of depression. Trends Immunol. 27, 24–31. 10.1016/j.it.2005.11.006 16316783 PMC3392963

[B110] Rajkowska G. O’Dwyer G. Teleki Z. Stockmeier C. A. Miguel-Hidalgo J. J. (2007). GABAergic neurons immunoreactive for calcium binding proteins are reduced in the prefrontal cortex in major depression. Neuropsychopharmacology 32, 471–482. 10.1038/sj.npp.1301234 17063153 PMC2771699

[B111] Rao Y. S. Mott N. N. Wang Y. Chung W. C. Pak T. R. (2013). MicroRNAs in the aging female brain: a putative mechanism for age-specific estrogen effects. Endocrinology 154, 2795–2806. 10.1210/en.2013-1230 23720423 PMC3713211

[B112] Reijmer Y. D. Brundel M. de Bresser J. Kappelle L. J. Leemans A. Biessels G. J.. (2013). Microstructural white matter abnormalities and cognitive functioning in type 2 diabetes: a diffusion tensor imaging study. Diabetes Care 36, 137–144. 10.2337/dc12-0493 22961577 PMC3526236

[B113] Revollo J. R. Korner A. Mills K. F. Satoh A. Wang T. Garten A.. (2007). Nampt/PBEF/Visfatin regulates insulin secretion in beta cells as a systemic NAD biosynthetic enzyme. Cell Metab. 6, 363–375. 10.1016/j.cmet.2007.09.003 17983582 PMC2098698

[B114] Rodgers J. T. Lerin C. Haas W. Gygi S. P. Spiegelman B. M. Puigserver P. (2005). Nutrient control of glucose homeostasis through a complex of PGC-1α and SIRT1. Nature 434, 113–118. 10.1038/nature03354 15744310

[B115] Rodriguez-Ortiz C. J. Baglietto-Vargas D. Martinez-Coria H. LaFerla F. M. Kitazawa M. (2014). Upregulation of miR-181 decreases c-Fos and SIRT-1 in the hippocampus of 3xTg-AD mice. J. Alzheimers Dis. 42, 1229–1238. 10.3233/JAD-140204 25024332 PMC7294908

[B116] Roiser J. P. Elliott R. Sahakian B. J. (2012). Cognitive mechanisms of treatment in depression. Neuropsychopharmacology 37, 117–136. 10.1038/npp.2011.183 21976044 PMC3238070

[B117] Rotella F. Mannucci E. (2013). Depression as a risk factor for diabetes: a meta-analysis of longitudinal studies. J. Clin. Psychiatry 74, 31–37. 10.1016/j.diabres.2012.11.022 23419223

[B118] Ruhe H. G. Mason N. S. Schene A. H. (2007). Mood is indirectly related to serotonin, norepinephrine and dopamine levels in humans: a meta-analysis of monoamine depletion studies. Mol. Psychiatry 12, 331–359. 10.1038/sj.mp.4001949 17389902

[B119] Russo S. J. Nestler E. J. (2013). The brain reward circuitry in mood disorders. Nat. Rev. Neurosci. 14, 609–625. 10.1038/nrn3381 23942470 PMC3867253

[B120] Sahin I. Alkan A. Keskin L. Cikim A. Karakas H. M. Firat A. K.. (2008). Evaluation of *in vivo* cerebral metabolism on proton magnetic resonance spectroscopy in patients with impaired glucose tolerance and type 2 diabetes mellitus. J. Diabetes Complicat. 22, 254–260. 10.1016/j.jdiacomp.2007.03.007 18413166

[B121] Schenk S. McCurdy C. E. Philp A. Chen M. Z. Holliday M. J. Bandyopadhyay G. K.. (2011). Sirt1 enhances skeletal muscle insulin sensitivity in mice during caloric restriction. J. Clin. Invest. 121, 4281–4288. 10.1172/JCI58554 21985785 PMC3204844

[B122] Schwer B. North B. J. Frye R. A. Ott M. Verdin E. (2002). The human silent information regulator (Sir)2 homologue hSIRT3 is a mitochondrial nicotinamide adenine dinucleotide-dependent deacetylase. J. Cell Biol. 158, 647–657. 10.1083/jcb.200205057 12186850 PMC2174009

[B123] Sekita A. Arima H. Ninomiya T. Ohara T. Doi Y. Hirakawa Y.. (2013). Elevated depressive symptoms in metabolic syndrome in a general population of Japanese men: a cross-sectional study. BMC Public Health 13: 862. 10.1186/1471-2458-13-862 24044502 PMC3848461

[B124] Shih J. Liu L. Mason A. Higashimori H. Donmez G. (2014). Loss of SIRT4 decreases GLT-1-dependent glutamate uptake and increases sensitivity to kainic acid. J. Neurochem. 131, 573–581. 10.1111/jnc.12942 25196144

[B125] Shimon H. Agam G. Belmaker R. H. Hyde T. M. Kleinman J. E. (1997). Reduced frontal cortex inositol levels in postmortem brain of suicide victims and patients with bipolar disorder. Am. J. Psychiatry 154, 1148–1150. 10.1176/ajp.154.8.1148 9247405

[B126] Silva N. Atlantis E. Ismail K. (2012). A review of the association between depression and insulin resistance: pitfalls of secondary analyses or a promising new approach to prevention of type 2 diabetes? Curr. Psychiatry Rep. 14, 8–14. 10.1007/s11920-011-0245-8 22094982

[B127] Silvestre M. F. Viollet B. Caton P. W. Leclerc J. Sakakibara I. Foretz M.. (2014). The AMPK-SIRT signaling network regulates glucose tolerance under calorie restriction conditions. Life Sci. 100, 55–60. 10.1016/j.lfs.2014.01.080 24530742

[B128] Smith D. L. Nagy T. R. Allison D. B. (2010). Calorie restriction: what recent results suggest for the future of ageing research. Eur. J. Clin. Invest. 40, 440–450. 10.1111/j.1365-2362.2010.02276.x 20534066 PMC3073505

[B129] Tennen R. I. Berber E. Chua K. F. (2010). Functional dissection of SIRT6: identification of domains that regulate histone deacetylase activity and chromatin localization. Mech. Ageing Dev. 131, 185–192. 10.1016/j.mad.2010.01.006 20117128 PMC2846990

[B130] Toups M. S. Myers A. K. Wisniewski S. R. Kurian B. Morris D. W. Rush A. J.. (2013). Relationship between obesity and depression: characteristics and treatment outcomes with antidepressant medication. Psychosom. Med. 75, 863–872. 10.1097/psy.0000000000000000 24163386 PMC3905462

[B131] Trivedi M. H. Greer T. L. (2014). Cognitive dysfunction in unipolar depression: implications for treatment. J. Affect Disord. 152–154, 19–27. 10.1016/j.jad.2013.09.012 24215896

[B132] van Duinkerken E. Schoonheim M. M. Ijzerman R. G. Klein M. Ryan C. M. Moll A. C.. (2012a). Diffusion tensor imaging in type 1 diabetes: decreased white matter integrity relates to cognitive functions. Diabetologia 55, 1218–1220. 10.1007/s00125-012-2488-2 22327286 PMC3296003

[B133] van Duinkerken E. Schoonheim M. M. Sanz-Arigita E. J. IJzerman R. G. Moll A. C. Snoek F. J.. (2012b). Resting-state brain networks in type 1 diabetic patients with and without microangiopathy and their relation to cognitive functions and disease variables. Diabetes 61, 1814–1821. 10.2337/db11-1358 22438575 PMC3379683

[B134] Vaziri H. Dessain S. K. Ng Eaton E. Imai S. I. Frye R. A. Pandita T. K.. (2001). hSIR2(SIRT1) functions as an NAD-dependent p53 deacetylase. Cell 107, 149–159. 10.1016/S0092-8674(01)00527-X 11672523

[B135] Wang Q. Liu M. Liu W. W. Hao W. B. Tashiro S. Onodera S.. (2012). *In vivo* recovery effect of silibinin treatment on streptozotocin-induced diabetic mice is associated with the modulations of Sirt-1 expression and autophagy in pancreatic beta-cell. J. Asian Nat. Prod. Res. 14, 413–423. 10.1080/10286020.2012.657180 22423887

[B136] Wang X. Guan Q. Wang M. Yang L. Bai J. Yan Z.. (2015). Aging-related rotenone-induced neurochemical and behavioral deficits: role of SIRT2 and redox imbalance and neuroprotection by AK-7. Drug Des. Devel. Ther. 9, 2553–2563. 10.2147/dddt.s81539 26089639 PMC4466888

[B137] Wong M. L. Dong C. Maestre-Mesa J. Licinio J. (2008). Polymorphisms in inflammation-related genes are associated with susceptibility to major depression and antidepressant response. Mol. Psychiatry 13, 800–812. 10.1038/mp.2008.59 18504423 PMC2650233

[B138] Xiao C. Kim H. S. Lahusen T. Wang R. H. Xu X. Gavrilova O.. (2010). SIRT6 deficiency results in severe hypoglycemia by enhancing both basal and insulin-stimulated glucose uptake in mice. J. Biol. Chem. 285, 36776–36784. 10.1074/jbc.m110.168039 20847051 PMC2978606

[B139] Yang H. Yang T. Baur J. A. Perez E. Matsui T. Carmona J. J.. (2007). Nutrient-sensitive mitochondrial NAD^+^ levels dictate cell survival. Cell 130, 1095–1107. 17889652 10.1016/j.cell.2007.07.035 PMC3366687

[B140] Ye J. Liu Z. Wei J. Lu L. Huang Y. Luo L.. (2013). Protective effect of SIRT1 on toxicity of microglial-derived factors induced by LPS to PC12 cells via the p53-caspase-3-dependent apoptotic pathway. Neurosci. Lett. 553, 72–77. 10.1016/j.neulet.2013.08.020 23973301

[B141] Yuen E. Y. Liu W. Karatsoreos I. N. Feng J. McEwen B. S. Yan Z. (2009). Acute stress enhances glutamatergic transmission in prefrontal cortex and facilitates working memory. Proc. Natl. Acad. Sci. U S A 106, 14075–14079. 10.1073/pnas.0906791106 19666502 PMC2729022

[B142] Zanoveli J. M. de Morais H. da Silva Dias I. C. Schreiber A. K. de Souza C. P. da Cunha J. M. (2015). Depression associated with diabetes: from pathophysiology to treatment. Curr. Diabetes Rev. 10.1159/000319510 25981499

